# Non-Identity of Croup and Diphtheria

**Published:** 1882-03

**Authors:** T. S. Latimer

**Affiliations:** Prof. of Physiology and Diseases of Children, College of Physicians and Surgeons, Baltimore


### ORIGINAL COMMUNICATIONS.

### THE NON-IDENTITY OF CROUP AND DIPHTHERIA.

By T. S. LATIMER, M. D.,
Prof. of Physiology and Diseases of Children, College of Physicians and Surgeons, Baltimore.

The researches of Hueter, Oertel and Buhl of Germany, appear at first sight to have established a causal relation between the spherical bacterium, or micrococcus and diphtheria, and the frequent coincident appearance of micro-bacteria in smaller numbers appears also to associate it with the development of diphtheritic false membrane, but the very frequent appearance of these organisms in parts unaffected with false membrane, and unattended by the constitutional state characteristic of diphtheria, compels us, I think, however reluctantly, to abandon the idea that the specific germ of diphtheria has at last been determined. Nevertheless I am of the opinion that though we cannot point to an organism characterizing diphtheria which is absent in pseudo-membranous croup, that there are differences, not only in the history and symptoms of these two diseases, but likewise an essential difference in the false membrane itself.

Rokitansky says of the croupous membrane that it is fibrinous exudation scarcely ever affecting any part but the air passages. He says: "The exudations present great differences in thickness and consistence; the membrane sometimes resembles an investment of hoar frost, or gauze, while at other times it will even exceed a line in thickness, while the consistence may vary from that of viscid cream to that of the most tough, compact, leathery, coagulated fibrin. But neither the density nor the consistency is uniform throughout; the exudation as a general rule becomes thinner and gradually softer towards its edges, more puriform or creamy, and the portion in contact with the mucous membrane is the softer and looser of the two." He also states that the croupous exudates are imperfectly adherent to the exudation surface. Let us place this excellent description of croupous membrane in sharp contrast with the no-less excellent description of diphtheritic membrane given by Rindfleisch, who says, "That which microscopically characterizes it, and has become the occasion of placing it as a membranous inflammation is the formation of a whitish-gray, compact, felted membrane which is elevated perhaps to the extent of half a line above the level of the mucous membrane, but *penetrates just as deep* into the substance of the mucous membrane and is the most intimately connected with it. The membrane is nothing that is *superposed*, nothing *secreted*, but the mucosa itself, as far as it has been partly tumefied, partly rendered anemic, even by the excessive infiltration with cells.

"This condition has, not improperly, been compared with a mortification by a chemical agent, with a corrosion, and the diphtheritic membrane has been designated a diphtheritic scab ; in fact the diphtheritic membrane is a caput mortuum, it can undergo no other changes than those of putrefaction, of decomposition."

We have here, I think, in striking contrast, the essential differences between the membranes. The one—croupous, is a fibrinous exudation; the other—diphtheritic, is nothing "superimposed, nothing secreted" but the mucosa itself. The one varies in consistence from a more or less compact membrane to a "creamy" consistence in the part last formed, or beneath the under surface; the other—diphtheritic—is a "compact felted membrane." The one sometimes separates entirely and is at all times but loosely attached at its last formed part—who of us that has performed tracheotomy in croup that has not seen the inferior extremity of membrane lifted with each expiratory gust, and sometimes thrust out at the opening? The other can only be separated by "putrefaction or decomposition." But there are other differences in the membranes, nor does this much rest on the statement of two individuals. Dr. Satterthwaite, of New York, in post mortems of diphtheria made for Dr. J. Lewis Smith, says "the boundary line between the false membrane and mucous surface could not be distinguished in many cases by the microscope, the net-work of pseudo-membrane extending into the mucous membrane; but here and there a line of separation could be made out; the pseudo and mucous surfaces being separated by a collection of *embryonal cells*."

Dr. Heitzman, of New York, reports in one case the mucous membrane intensely injected, but "without any membranous exudation."

Dr. J. Lewis Smith distinguishes between the membrane occurring in the trachea and bronchial tubes, where he says it can be readily detached; and the same membrane in the larynx and superjacent parts where he says "it penetrates the entire mucous membrane," and can not be distinguished from it. Dr. Day, of London, in the last edition of his book on diseases of children, remarks: "The false membrane formed in the larynx in genuine cases of croup is quite different from the leathery or yellowish-gray exudation, found on the tonsils in the larynx and bronchial tubes in cases of diphtheria;" but he does not state in what the difference consists.

Green, in his "Pathology and Morbid Anatomy," finds it often difficult to draw any line of demarkation between the histological changes occurring in diphtheria and those of croup; but finds the submucous tissue usually more deeply involved in diphtheria.

Professor Steiner, of the University of Prague, says tersely: "True croupous exudation is free and membranous, whilst the diphtheritic is parenchymatous, with necrobiosis."

The necrobiotic tendency is the distinguishing characteristic of diphtheritic false membrane, and rarely, if ever, is found to characterize croupous membrane.

In Reynolds' System of Medicine, Dr. Squire says: "The false membrane of Croup differs from that formed during the specific inflammation, both in its chemical and physiological relations."

Wagner declares that the croupous membrane contains no fibrin, but consists of epithelial cells in process of degeneration, which, as they are pushed forward from the mucous surface, enlarge and come to resemble irregular blocks interlacing with each other; but more recent investigations by Rindfleisch, Delafield, Carl Weigert and others, demonstrate the presence of fibrin which Rindfleisch holds occurs in layers, alternating with layers of cells forming a stratified membrane of from one to ten layers.

Weigert "concludes that the undermost part of the croupous layers consists of epithelial debris. Secondly, immediately above this he found a different layer, consisting of a net work of delicate fibres, in the meshes of which were free nuclei. This net work evidently consists of fibrin, as it gave the reactions of this substance. Thirdly, penetrating the upper part of the fibrinous net-work, and overlying it was a layer of mucus containing large cells with large nuclei and grains of black pigment. From all these examinations which have been made by competent microscopists, we must conclude that this croupous exudation consists largely of altered epithelial cells, and that it also contains a network of fibrin."*

Let us suppose, however, for the sake of argument that these alleged differences do not exist, and that the false membrane of croup

*Lewis Smith, Diseases of Children.

and diphtheria are indistinguishable, and endeavor to see what other things distinguish these affections.

First, The site of the local trouble: Rokitansky, as we have already seen, says the croupous membrane never attacks any locality but the air passages, and in this opinion most authorities of whom I have knowledge agree ; whereas the diphtheritic false membrane may, and frequently does appear on any inflamed surface, a blister, a wound, an old ulcer on any part of the body, though showing a decided preference for the pharyngeal and laryngeal mucous membranes.

In diphtheria the pharynx rarely, if ever, escapes, indeed the local trouble almost invariably begins there. I have never known a case in which the pharynx was not first affected.

In croup the pharynx is usually exempt, the larynx alone being affected. I have never known a case of croup involving the pharynx.

Dr. Squire asserts positively that in diphtheria some part of the pharynx is sure to be affected; and quotes from Dr. Cheyne: "In other diseases, inflammation and incrustation extending from the fauces to the larynx, may often be observed, but in genuine croup, as it exists in Scotland and in Ireland, never."

Dr. Day also says briefly: "Diphtheria generally begins in the pharynx, croup in the larynx." In Guy's Hospital Reports for 1877, Drs. Lamb and Hilton Fagge state that it must be admitted that diphtheria is capable of occasioning plastic inflammation in the larynx without affecting the pharynx; but that the cases in which this occurs are "in the highest degree exceptional."

The testimony to this effect is so general that it seems unnecessary to cite authorities.

False membrane may occur in the larynx from many causes. It has been experimentally induced many times. Dr. Murchison found it a frequent complication of typhoid fever. It occasionally complicates small-pox; it occurs in measles. We all have seen it intercurrent in scarlatina, Indeed, I am strongly convinced that it is a lesion of frequent occurrence in most constitutional affections, characterized by degenerative blood changes, and that in each of these, not excepting diphtheria, it is an accident of the disease rather than a distinctive lesion. In croup alone—except when experimentally induced—it is *the* disease itself. In all other diseases the dyspnœa indicating its presence is secondary to other symptoms. In croup it is primary and alone, except from symptoms plainly referable to respiratory obstruction.

In all other diseases death may occur without the development of false membrane; in croup alone it is the manifest cause. In all other diseases in which it is present death may occur after entire relief from the respiratory trouble; in croup rarely, if ever. Other diseases, especially diphtheria, either through the direct action of their several specific poisons or through profound changes wrought in the blood or nervous system show lesions in kidney, spleen or other organs sufficient to account for the fatal result; croup never.

The diphtheritic false membrane appears to contain the specific element by which the disease may be propagated, as, I doubt not, does also the membrane occurring in small-pox, measles, scarlatina, or typhoid fever.

Though I am unaware of any experiments bearing on this point, I am convinced croup could not be so propagated except it were introduced into the laryngeal mucous membrane; and then, if at all, only through the local irritation, set up as when experimentally induced by application of ammonia or other agents.

My reason for thinking so is the extreme rarity with which this affection attacks two members of the same family. I have never seen a case in which the larynx was solely or primarily affected, as is characteristically the case in croup, in which I thought it necessary to isolate the patient; nor have I ever under such circumstances known it to be communicated to others, although I admit that diphtheria may possibly first affect the larynx.

In diphtheria the superficial lymphatic glands in the cervical region are almost invariably affected. In croup never.

In diphtheria there is a distinct incubative period, (2 to 5 days, Oertel) after exposure to infection. In croup (meaning by this term, case where larynx is primarily affected by diphtheria cases in which it is secondarily affected) there is a history of exposure to draughts and other ordinary causes of cold quickly followed by laryngeal disturbance.

Diphtheria occurs at all ages. Croup is essentially a disease of childhood. Finally: Diphtheria is followed by affections scarcely less grave than the primary trouble. Diphtheritic paralysis affecting any or all of the muscles of the body, including those of bladder and rectum; valvular diseases of the heart and tubular nephritis; and in most severe cases, prolonged convalescence. Croup is practically free from sequelæ, and convalescence is rapid.

The result of the discussion in the Royal Medical and Chirurgical Society in 1879, says Dr. Day: "was that membranous laryngitis may arise from common inflammation, or in connection with specific disorders of various kinds, but that the most frequent cause is diphtheria;" and this appears to me to be the essence of the whole matter. I can see no more propriety in speaking of croup and diphtheria as one, than in speaking of typhoid fever and diphtheria as one, because they may each have developed a laryngeal false membrane, except that the membrane is more uniformly present in diphtheria than in typhoid. I am also convinced that in a very large proportion of cases of diphtheria, there is no extension of the trouble to the larynx.

